# Biomechanical Analysis of the Jump Shot in Basketball

**DOI:** 10.2478/hukin-2014-0062

**Published:** 2014-10-10

**Authors:** Artur Struzik, Bogdan Pietraszewski, Jerzy Zawadzki

**Affiliations:** 1 Department of Biomechanics, University School of Physical Education, Wrocław, Poland.

**Keywords:** basketball, jump shot, countermovement jump, power, soft landing

## Abstract

Basketball players usually score points during the game using the jump shot. For this reason, the jump shot is considered to be the most important element of technique in basketball and requires a high level of performance. The aim of this study was to compare the biomechanical characteristics of the lower limbs during a jump shot without the ball and a countermovement jump without an arm swing. The differences between variables provide information about the potential that an athlete can utilise during a game when performing a jump shot. The study was conducted among 20 second-league basketball players by means of a Kistler force plate and the BTS SMART system for motion analysis. The variables measured included the take-off time, mean power, peak power, relative mean power, jump height, maximum landing force and calculated impact ratio. Surprisingly, more advantageous variables were found for the jump shot. This finding suggests a very high performance level in the jump shot in the studied group and a maximum utilisation of their motor abilities. Both types of jumps were characterised by high mean and peak power values and average heights. The high forces at landing, which result in considerable impact ratios, may have prompted the studied group to land softly. Use of the countermovement jump without an arm swing is recommended to assess and predict the progression of player’s jumping ability.

## Introduction

The main objective of each basketball player during a game is to score points. In an attempt to do so, an athlete might perform a jump shot, set shot, layup or a free throw. As the discipline has evolved and more athletic players have practised this sport discipline, defence has become increasingly efficient. As a result, the two-legged jump shot has become more frequent, amounting to over 70% of all the shots during a game, which necessitates a greater performance level for athletes executing the jump shot to increase the height at which the ball is released (i.e., the release point) (Oudejans et al., 2012). This movement must be automated so that, regardless of the external factors, the player achieves maximum repeatability (Kornecki et al., 2002). The factors that affect the height at which a shot is performed include the shooter body height, jump height and arrangement of body parts (Miller and Bartlett, 1996). When a player is covered by an aggressive defender, his aim is to perform the shot at the highest possible release point. Additionally, the shot must reach that release point in the shortest time frame. These factors result in an extension of the body in players performing the jump shot (Rojas et al., 2000).

Previous biomechanical studies on shots performed by basketball players have typically measured kinematic variables, such as maximum angular values and angular velocities in players’ individual joints (McClay et al., 1994a), temporal profiles of changes in the angular values of individual joints and angular velocities (Kornecki and Lenart, 1997), the release angle and velocity of the ball, changes in the location and velocity of the centre of mass, the rotation of the upper body of a shooter (Miller and Bartlett, 1996), the effect of initial ball rotation (Tran and Silverberg, 2008) and the foot position when shooting (Spina et al., 1996). Therefore, the focus of prior studies was on the shooting technique rather than the motor abilities of the shooter. In-depth analyses of the free throw have also been conducted (Tran and Silverberg, 2008).

An analysis of the ground reaction forces generated by basketball players during a shot provides information concerning the phases of take-off and landing (McClay et al., 1994b), which permits the studies to focus on not only the jumping performance (i.e., the jump height and time to reach this height, indirectly represented by the power of the lower limbs), which is of key importance to the shot, but also on the health-oriented aspects of this movement. Learning a soft landing technique is essential for basketball players, despite the use of well-cushioned footwear (Brizuela et al., 1997). Hard landing causes an excessive load on the lower limbs (which may potentially exceed the body weight by several times), which in effect can lead to local overload and injuries. Therefore, it is important that a player absorbs the shock (by flexing his lower limbs during landing) and does not land on extended legs. An earlier contact of the heel with the ground increases the impact force, which is why midfoot landing is preferred over landing with the whole foot (DeVita and Skelly, 1992; Bober et al., 2002).

The shooting techniques of different basketball players seem similar, although the differences are sufficiently significant to the point that each player can be considered to have a unique shooting style. This observed phenomenon is due to different length proportions between upper body segments (Kornecki et al., 2002). Furthermore, a shooting technique (often resulting from a particularity of external factors, e.g., situation shots) is typically justified by its accuracy. Therefore, it is more reasonable to place emphasis on variables responsible for the release height than on the minute technical nuances of the shot. Training that focuses on maximising the release height will allow for coping with a defender and performing a shot from a more convenient position. A high level of performance in a jump shot forces players to utilise their maximum jumping (i.e., speed-strength) and coordination abilities. A motor ability particularly important to a basketball player is the power generated by the lower limbs, as the game of basketball is based on explosive movements, such as accelerations, quick cuts and jumps.

The arm swing executed prior to take-off helps reach greater jump heights in the countermovement jump (CMJ) (Hara et al., 2006). However, when performing a jump shot, basketball players use their upper limbs in ways that do not necessarily enhance their jumping performance, i.e., motions other than arm swings. Additionally, it is not known whether this upper limb movement is a factor that affects the utilisation of maximum abilities of the lower limbs during a jump. A classical measure of maximum speed-strength abilities of lower limbs when performing a vertical jump is the CMJ without an arm swing. Therefore, the lower limb power and the jump height will be smaller in the jump shot. Thus, the aim of the present study was to compare biomechanical characteristics of lower limbs (in take-off and landing phases) achieved by a basketball player when performing a jump shot and the maximum CMJ achieved without an arm swing. The potential differences between these two movements are likely to provide information about the additional potential a player might utilise during a game when performing a jump shot. Can a player achieve similar values of lower limb variables in the jump shot and in the CMJ without an arm swing? If so, that player would demonstrate the ability to perform one of the most important technical elements of basketball.

## Material and Methods

The study was conducted among 20 well-trained junior division II basketball players. The study group was characterised by the following mean properties (±SD): body height – 193.1 ± 7.9 cm, body mass – 84.8 ± 9.8 kg, age – 18.4 ± 1.3 years. In all measurements, the subjects wore professional basketball footwear typically used during training and competition. The tests were performed in a Biomechanical Laboratory. Prior to the tests, the participants were familiarised with the purpose of the study. Additionally, each participant provided their written consent for participation in the experiment. The research project was approved by the Senate’s Research Bioethics Commission at the University School of Physical Education in Wrocław, Poland.

To measure ground reaction forces, one Kistler force plate (model: 9286A; Winterthur, Switzerland) with BioWare® software was used for each leg. Displacements of the upper level of the lower limbs were measured with the BTS SMART system (BTS Bioengineering, Milan, Italy) using passive markers that reflect the emitted infrared radiation (IR). The markers were located at the greater femoral trochanters on both sides of the body. The system used 6 cameras to capture at a frame rate of 120 Hz and 0.2 mm resolution. To synchronise the measurements, the sampling frequency of the platforms was set at 240 Hz. The BTS-SMART Analyzer software was used to facilitate the synchronisation of the recorded data.

Prior to the examination, each subject warmed up for 10 minutes. Athletes were also given the opportunity to practice the movements until they felt they were fully prepared. Each subject performed a maximum CMJ without an arm swing (i.e., hands resting on hips) and a jump shot without the ball (Picture 1) according to the following instruction: ‘Perform the jump shot as if you were holding a ball standing 6 metres from the basket in front of an opponent who is standing in a defensive position with his upper extremity extended’. Performing the arm swing with the upper limbs prior to the take-off leads to a higher jumping height. Therefore, both movements studied were performed without an arm swing (Hara et al., 2006).

Countermovement and take-off phase times were calculated using BioWare® 5.1.1.0 software. The separation between the countermovement and take-off phases (i.e., when the impulse is zero) was determined based on the integration of the vertical component of the ground reaction forces (reduced by the weight of the subject) with respect to time. The power of the lower limbs was calculated as the product of the vertical ground reaction force (recorded by force plates) and velocities of the movement of lower limb extension as represented by the vertical velocity of the markers located on the greater femoral trochanters (recorded by the BTS Smart system based on the displacement of the markers). The relative mean power (*P_ju_*) was calculated based on the equation used in a study by Pietraszewski and Rutkowska-Kucharska (2011):

$$P_{\mathrm{ju}}=\frac{gh_{s}}{t_{o}},$$

where *h_s_* is the jumping height, *t_o_* is the take-off time, and *g* is the acceleration due to gravity.

The jump height (*h_s_*) was calculated based on the time of the flight phase (*t_l_*):

$$h_{s}=\frac{1}{\alpha }gt_{l}^{2}.$$

The impact ratio (IR) is the quotient of maximum values of ground reaction forces during landing (*F_l_*) and take-off (*F_o_*) phases:

$$\text{IR}=\frac{F_{l}}{F_{o}}.$$

To analyse the differences between individual variables, the authors used a *t*-test to determine the significance of differences for dependent variables due to the normality of the distribution of the variables. The level of significance was set at *α* = 0.05.

## Results

**Figure 1:**
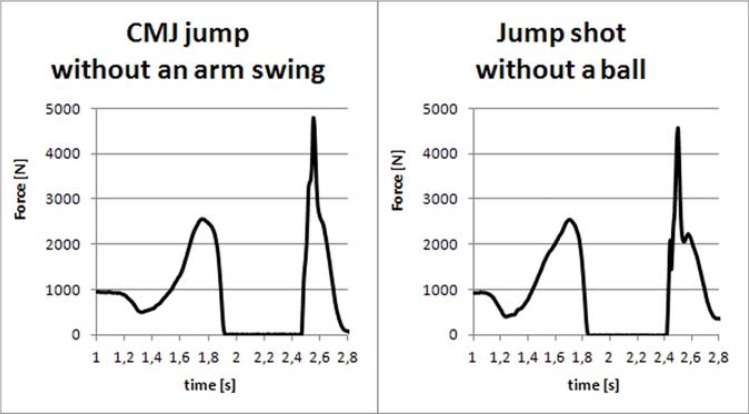
Ground reaction forces during a maximum CMJ jump without an arm swing and a jump shot without a ball

**Figure 2:**
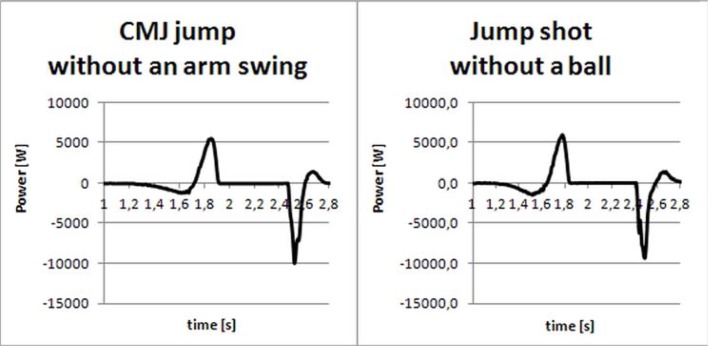
Courses of the power during a maximum CMJ jump without an arm swing and a jump shot without a ball

For both studied movements, similar curve profiles of *F(t)* and *P(t)* (Figures 1 and 2) justified the jump shot without the ball assessment by the CMJ without an arm swing. Table 1 presents mean values (±SD) of the variables obtained in both the jump shot and the maximum CMJ without an arm swing. Table 2 shows the comparison between the jumps. The values are the ratios of the variables obtained in the jump shot and their counterparts obtained with the maximum CMJ without an arm swing. For the take-off time (*t_o_*), the dividend and divisor values were switched to obtain uniform values. In Table 2, each value greater than 1 indicates an advantage of the jump shot over the jump without an arm swing for that particular variable; conversely, each value less than 1 indicates an advantage of the jump without an arm swing over the jump shot for that particular variable. The average maximum ground reaction forces in the landing phase were 5.57 ± 1.22 multiplied by the average body weight for the jump shot and 5.39 ± 1.3 for the CMJ without an arm swing. The statistical analysis revealed no significant differences between the heights of both types of jumps. All other variables in Table 1 were significantly different from each other (*p* < 0.05).

## Discussion

The mean values presented in Table 2 indicate that when performing the jump shot, basketball players had improved take-off times and peak powers and an overall improved mean power in the take-off phase and relative mean power. The only variable that was not significantly greater relative to its average value in the maximum CMJ without an arm swing was the jump height, although differences were observed in only seven subjects. Therefore, it can be assumed that the jump heights in both motions were the same. However, due to their training as highly skilled basketball players, the jump shot was a movement that the subjects performed well, allowing for the maximum utilisation of their speed-strength abilities. It is unlikely that similar results would be observed in other subjects.

The basketball players had a lower impact ratio in the jump shot (i.e., a lower value of the ratio between the landing force and the take-off force). Nevertheless, the values of this variable were high in both motions (i.e., the landing force was over twice the take-off force), which could lead to injuries. Additionally, the mean landing force was more than five times greater than the body weight. Similar problems with improper landing were also observed in professional US National Basketball Association (NBA) players. In particular, among NBA players, a higher landing force but a lower impact ratio were observed in the jump shot compared with the vertical jump (McClay et al., 1994b).

The basketball players in this study had higher values of relative mean power compared with those of athletes (also including basketball players) studied by Pietraszewski and Rutkowska-Kucharska (2011) who performed CMJs with arm swings; the jump heights in both cases were similar. Furthermore, in two studies by Buśko (1988; 1989), basketball players performing CMJs with arm swings exhibited significantly lower values of peak power in the lower limbs, but resulting in approximately 10 cm greater jump height. However, the arm swing motion has a positive effect on increasing the vertical jump height (Hara et al., 2006). The basketball players tested by Schiltz et al. (2009) had similar results for jump height without an arm swing as those of the basketball players in this study. Furthermore, basketball players studied by Alemdaroğlu (2012) reached lower heights for both CMJ and SJ (squat jump) motions. In another study, after a variety of plyometric regimens, a group of youth basketball players initially obtained similar vertical jump heights, and after 10 weeks, the heights increased by approximately 5 cm (Kukrić et al., 2012). In a study conducted by Shallaby (2010), similar initial heights were observed in basketball players subjected to 12 weeks of plyometric training, and jumping heights increased by 40 to 70%. However, the most interesting comparison was observed in the study by Boraczyński and Urniaż (2008) who tested a group of basketball players after an 8-week plyometric regimen. Prior to the regimen, the players had comparatively worse take-off times and peak powers when executing CMJs. Furthermore, greater values were measured for jump height, mean power during the take-off phase and relative mean power. Despite improvements in all jumping variables after the training cycle, the mean value of the take-off time and the peak power remained worse compared with the basketball players analysed in this study.

When comparing two jumps with similar maximum height, the ratio of the variables measured should be almost identical. The authors recommend the CMJ without an arm swing as a useful tool in predicting the progression of the jumping ability of a basketball player in executing the jump shot, both in terms of the absolute (i.e., the jump height) and relative performance (i.e., the power of lower limbs). Specialised test procedures using force plates (and a system for motion analysis) allow coaches to collect information about the motor reserves in a player and the effectiveness of his potential. These data can be useful in constructing individualised training regimens for athletes. The variables presented in this study, which describe the jump without an arm swing and the jump shot, can also be measured via the ground reaction forces (without requiring a motion analysis system). However, the estimated power measurement errors of the lower limbs would likely be at least 2.6–3.5% greater.

## Conclusion

1. The mean values for take-off time, mean power, peak power and relative mean power were greater in the jump shot. No statistically significant differences were observed between jump heights in the jump shot and the CMJ without an arm swing.
2. The movement of the upper limbs in the jump shot does not reduce the jumping performance. With an adequate level of coordination, this consistency allows a player to fully utilise the speed-strength abilities of their lower limbs in the jump shot.
3. The CMJ without an arm swing may be a good measure of the jumping abilities of basketball players when performing the jump shot.
4. High impact ratios and landing forces suggest the necessity of a greater emphasis on soft landing in basketball training.

## Figures and Tables

**Picture 1:**
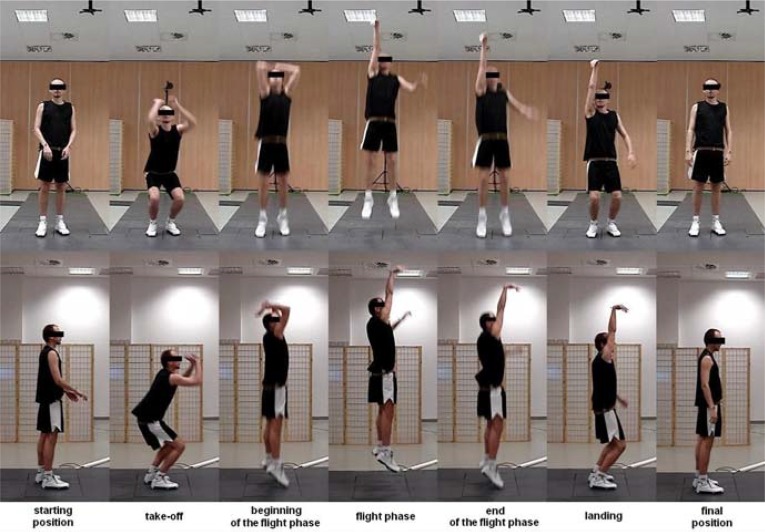
A jump shot without a ball

**Table 1:** Mean values ±SD of take-off time (t_o-s_), mean power during take-off phase (P_s_) and relative mean power (P_ju-s_) calculated separately for either lower limb in part A and peak power (P_max-s_), jump height (h_s-s_) and impact ratio (IR_s_) calculated for both lower limbs in part B, for jump shot; and mean values ±SD of take-off time (t_o-cmj_), mean power during take-off phase (P_cmj_) and relative mean power (P_ju-cmj_) calculated separately for either lower limb in part A and peak power (P_max-cmj_), jump height (_hs-cmj_) and impact ratio (IR_cmj_) calculated for both lower limbs in part B, for maximum CMJ without an arm swing

		part A			part B		
	
		*t_o-s_* (s)	*P_s_* (W)	*P_ju-s_* (W/kg)	*P_max-s_* (W)	*h_s-s_* (m)	IR*_s_*
	
Jump shot	Left limb	0.18 ± 0.03	1757.3 ± 430.3	21.1 ± 5.5	4836.9 ± 565.9	0.365 ± 0.06	2.04 ± 0.5
	Right limb	0.18 ± 0.03	1497.9 ± 646.9	20.5 ± 5			

CMJ without an arm swing		*t_o-cmj_* (s)	*P_cmj_* (W)	*P_ju-cmj_* (W/kg)	*P_max-cmj_* (W)	*h_s-cmj_* (m)	IR*_cmj_*

	Left limb	0.22 ± 0.04	1415.2 ± 325.2	16.9 ± 3.6	4391.7 ± 574.6	0.368 ± 0.045	2.26 ± 0.66
	Right limb	0.22 ± 0.05	1215.8 ± 554.9	16.9 ± 4.1			

**Table 2:** Mean values ±SD that compare individual variables of the jump shot and a maximum CMJ without an arm swing

	*t_o-cmj_/t_o-s_*	*P_max-s_/P_max-cmj_*	*P_s_/P_cmj_*	*P_ju-s_/P_ju-cmj_*	*h_s-s_/h_s-cmj_*
Left limb	1.29 ± 0.28	1.11 ± 0.11	1.27 ± 0.28	1.27 ± 0.28	0.99 ± 0.12
Right limb	1.25 ± 0.27		1.24 ± 0.32	1.24 ± 0.32	
